# The physiological sonographic features of the ovary in healthy subjects: a joint systematic review and meta-analysis by the Italian Society of Gynecology and Obstetrics (SIGO) and the Italian Society of Endocrinology (SIE)

**DOI:** 10.1007/s40618-022-01939-8

**Published:** 2022-11-24

**Authors:** F. Moro, I. Scavello, E. Maseroli, G. Rastrelli, C. Baima Poma, C. Bonin, F. Dassie, S. Federici, S. Fiengo, L. Guccione, M. Villani, A. Gambineri, R. Mioni, P. Moghetti, C. Moretti, L. Persani, G. Scambia, F. Giorgino, L. Vignozzi, the Women’s Endocrinology Group of the Italian Society of Endocrinology, A. Gambineri, R. Mioni, P. Moghetti, P. Moretti, L. Persani, L. Vignozzi

**Affiliations:** 1 Dipartimento Scienze della Salute della Donna, del Bambino e di Sanità Pubblica, Fondazione Policlinico Universitario Agostino Gemelli, IRCCS, Rome, Italy; 2 Department of Experimental Clinical and Biomedical Sciences “Mario Serio”, University of Florence, Viale Pieraccini 6, 50134 Florence, Italy; 3 Andrology, Women’s Endocrinology and Gender Incongruence Unit, Azienda Ospedaliero-Universitaria Careggi, Florence, Italy; 4 Consultorio Familiare ASL Città di Torino, Turin, Italy; 5 Unit of Obstetrics and Gynecology B, Department of Women and Children’s Health, AOUI Verona, Verona, Italy; 6 Department of Medicine, Clinica Medica 3-Azienda Ospedaliera, University of Padua, Padua, Italy; 7 Unit of Andrology and Reproductive Endocrinology, Department of Endocrine and Metabolic Diseases, IRCCS Istituto Auxologico Italiano, 20149 Milan, Italy; 8 Department of Obstetrics and Gynaecology, ARNAS Civico Hospital, Palermo, Italy; 9 Department of Systems’ Medicine, University of Tor Vergata, Rome, Italy; 10 Unit of Endocrinology, Diabetes and Metabolism, Department of Medicine, University of Verona, Verona, Italy; 11 Division of Endocrinology and Diabetes Prevention and Care, IRCCS Azienda Ospedaliero-Universitaria di Bologna, Bologna, Italy; 12 Department of Medical and Surgical Sciences (DIMEC), Alma Mater Studiorum University of Bologna, Bologna, Italy; 13 Department of Medical Biotechnology and Translational Medicine, University of Milan, 20121 Milan, Italy; 14 Istituto Di Clinica Ostetrica E Ginecologica, Università Cattolica del Sacro Cuore, Rome, Italy; 15 Section of Internal Medicine, Endocrinology, Andrology and Metabolic Diseases, Department of Emergency and Organ Transplantation, University of Bari Aldo Moro, Bari, Italy

**Keywords:** Ovary, Ultrasound, Ovarian volume, Follicular count

## Abstract

**Purpose:**

There is a lack of uniformity in the definition of normal ovary ultrasound parameters. Our aim was to summarize and meta-analyze the evidence on the topic. Full-text English articles published through December 31, 2020 were retrieved via MEDLINE and Embase. Data available for meta-analysis included: ovarian follicular count, ovarian volume, and ovarian Pulsatility Index (PI) assessed by Doppler ultrasound.

**Methods:**

Cohort, cross-sectional, prospective studies with a single or double arm were considered eligible. Interventional studies were included when providing baseline data. Both studies on pre- and post-menopausal women were screened; however, data on menopausal women were not sufficient to perform a meta-analysis. Studies on pre-pubertal girls were considered separately. Eighty-one papers were included in the meta-analysis.

**Results:**

The mean ovarian volume was 6.11 [5.81–6.42] ml in healthy women in reproductive age (5.81–6.42) and 1.67 ml [1.02–2.32] in pre-pubertal girls. In reproductive age, the mean follicular count was 8.04 [7.26–8.82] when calculated in the whole ovary and 5.88 [5.20–6.56] in an ovarian section, and the mean ovarian PI was 1.86 [1.35–2.37]. Age and the frequency of the transducers partly modulated these values. In particular, the 25–30-year group showed the higher mean follicular count (9.27 [7.71–10.82]), followed by a progressive age-related reduction (5.67 [2.23–9.12] in fertile women > 35 years). A significant difference in follicular count was also found according to the transducer’s upper MHz limit.

**Conclusion:**

Our findings provide a significant input to improve the interpretation and diagnostic accuracy of ovarian ultrasound parameters in different physiological and pathological settings.

## Introduction

Ultrasound examination is the standard imaging method to analyze ovarian morphology, while providing also some important functional information or to identify patients with polycystic ovary morphology (PCOM) [1]. The existing Rotterdam guidelines define the ultrasound characteristics of PCOM by the presence of ovarian volume > 10 ml or the presence of 12 or more follicles measuring 2–9 mm in diameter in each ovary [1]. Other typical ultrasound features of PCOM have been widely studied including central stromal echogenicity [2], increased ovarian blood flow [reduced pulsatility index (PI) and reduced resistance index] [3], stromal index and stromal to ovarian area ratio [4, 5]. In addition, the Androgen Excess Society guidelines criteria have increased the threshold count of small ovarian follicles to 25 [6]. However, the accurate determination of numerous follicles can be obtained only with the new-generation US machines, not available in many centers.

The structure of the ovary is basically made up of an outer cortical and an inner medullary region. The cortex consists primarily of follicles in different stages of maturation, the medulla of stromal cells, lymphatics, blood vessels, and nerves. The sonographic features of the ovaries are highly variable, depending on the cyclic influence of the hypothalamic–pituitary hormonal axis, which determines ovarian hormone production, follicular maturation, and degeneration [7].

Ultrasound examination is also an excellent diagnostic tool to discriminate between benign and malignant ovarian masses in the hands of experienced examiners using subjective assessment [8]. A consensus opinion on terms, definitions, and measurements to describe the sonographic features of adnexal tumors was established by the International Ovarian Tumor Analysis (IOTA) Group [9]. The IOTA group created ultrasound-based models with similar accuracy to that of expert ultrasound examiners to characterize the ultrasound appearance of benign ovarian tumors (i.e., endometriomas, dermoid cysts, cystadenofibromas) [10–12], and to differentiate from the borderline [13, 14], and the malignant ones [15–17].

In contrast, the sonographic appearance of the ovary in physiological conditions has been poorly investigated. Only some prospective studies described ovarian characteristics in healthy pre- and post-menopausal women in terms of volume and vascularization patterns [18–20]. However, there is a lack of uniformity in the definition of normal ovary ultrasound parameters and no consensus statement has been established.

Two Italian societies—the Italian Society of Gynecology and Obstetrics (SIGO) and the Italian Society of Endocrinology (SIE)—agreed on the urgent need to produce a consensus statement to define normal ovary ultrasound parameters. To reach this goal, a joint commission of the 2 societies promoted a systematic review and meta-analysis of the existing evidence on ultrasound parameters of the normal ovary. Therefore, the aim of this review is to define the sonographic parameters of the normal ovary, including ovarian follicular count, ovarian volume, and vascular indices. This represents a fundamental and critical step for orientating clinicians not only in interpreting sonographic data, but also to substantiate future research in the field of pathological conditions such as Polycystic Ovary Syndrome (PCOS).

Even though majority of retrieved records enrolled pre-menopausal women, we did not exclude the few studies involving post-menopausal ones, and presented data accordingly.

## Methods

### Research question

This study aimed to answer this question: which are the normal ovarian follicular count, ovarian volume, ovarian stroma, and vascular indices assessed by ultrasound and Doppler ultrasound?

### Study outcomes

The study outcomes were the mean values of the following parameters: mean ovarian follicular count (whole ovary; number), mean ovarian follicular count (ovarian section; number), mean ovarian volume (ml), and mean ovarian artery PI (number). We were not able to provide a meta-analysis of ovarian stroma volume or other vascular indices [i.e., PSV (peak systolic velocity) and RI (Resistance Index)] due to the lack of an adequate number of eligible studies reporting these measures.

### Type of study design included

Studies that are considered eligible were cohort studies, cross-sectional studies, and prospective studies with a single arm or including two groups (i.e., healthy controls). We included interventional studies when they provided baseline data (obtained before any treatment, i.e., ovarian stimulation for Assisted Reproductive Technology, ART).

### Study population

Both studies on pre-menopausal and post-menopausal women were considered eligible for the qualitative analysis; however, data on menopausal women were not sufficient to perform a meta-analysis. Studies on pre-pubertal girls were considered separately. Records were selected when presenting data obtained from healthy volunteers (including control arms) or from the general population (i.e., screening studies). Regarding data on women from infertile couples, they were included only when a male factor or a tubal factor was specified as the only identified infertility factor.

### Search strategy

We performed a systematic review of the literature using methodological approaches previously published [21], and following a protocol written prior to starting the review (PROSPERO registration protocol: CRD42022300584). An extensive search was performed in the following databases: MEDLINE and Embase. Only articles in English and with full-text were included. The search was performed using the words “ovarian”, “Doppler” and “ultrasound” [All Fields], accruing all records on human beings published between January 2000 and December 31, 2020.

### Study selection

All the team members independently screened records for inclusion, blinded to each other’s’ decisions. Two of the team members (IS and EM) checked decisions and resolved eventual conflicts. Selections were recorded in a dedicated Excel spreadsheet.

### Data extraction

The following data were recorded: number of subjects, ovarian volume (ml; mean and standard deviation—SD), ovarian follicular fount (in the whole ovary or in the ovarian section, mean and SD), ovarian artery Pulsatility Index (PI) (mean and SD), reproductive stage of enrolled subjects, (pre-pubertal/reproductive age/menopausal), age, 2D (2 dimensional) or 3D ultrasound technique, use of transabdominal (TA) or transvaginal (TV) approach, menstrual phase in reproductive age women, description of the size of the follicles included in the follicular count, upper limit of the frequency of the transducer used in MHz, and localization of the artery sampled for Doppler analysis. All the team members independently extracted data and two of the team members (IS and EM) checked them. Disagreements between individual judgements was resolved by collective discussion during dedicated online meetings. Study investigators were contacted for unreported data or additional details. Data were recorded in a dedicated Excel spreadsheet.

### Quality assessment

The quality of studies was assessed according to the Oxford Centre for Evidence-Based Medicine: Levels of Evidence [22]. As shown in Table 1, the vast majority of studies were judged as Level 3 (non-randomized or cohort studies).

**Table 1:** Description of the studies included in the meta-analysis

Author [Ref.]	Year	LoE	Study design	No. of subjects	Age, years (mean)	Fertility status	Menstrual phase	Probe upper MhZ limit	Method	Follicles description	Follicles: section vs. whole ovary	Artery sampled for Doppler analysis (PI)
Adali et al. [24]	2009	3	Prospective	42	24.3	Reproductive age	Follicular	6.5	2D TV	NA	NA	Inside ovarian stroma, not close to surface or follicles
Adams et al. [25]	2004	2	Retrospective	29	28.4	Reproductive age	Follicular	5.0	2D TA and TV	2–8 mm	NA	NA
Ajossa et al. [26]	2002	3	Prospective	15	29.5	Reproductive age	Follicular	7.0	2D TV	2–8 mm	NS	NA
Alebić et al. [27]	2018	3	Retrospective	705	33.4	Reproductive age	follicular	7.0	2D TV	2–9 mm	Whole	NA
Allemand et al. [28]	2006	3	Retrospective	29	30.9	Reproductive age	Follicular	8.0	2D and 3D TV	< 10 mm	Whole/section	NA
Assens et al. [29]	2020	3	Prospective	115	16	Reproductive age	Follicular	8.0	2D and 3D TA	2–8 mm	Whole	NA
Aviram et al. [30]	2008	3	Retrospective	77	NR	Menopausal	NA	NR	2D TV	NA	NA	NA
Badouraki et al. [31]	2008	3	Prospective	99	6.9	Prepubertal	NA	7.5	2D TA	NA	NA	NA
Bancsi et al. [32]	2002		Prospective	120	34.9	Reproductive age	Follicular	7.5	2D TV	< 5 mm	NA	NA
Basir et al. [33]	2001	3	Prospective	20	33	Reproductive age	Follicular	7.0	2D TV	NA	NA	Within ovarian parenchyma, close proximity to follicles or c.l
Bath et al. [34]	2003	3	Retrospective	11	23	Reproductive age	Follicular	4.0	2D TV	2–10 mm	Whole	NA
Battaglia et al. (a) [35]	2002	3	Prospective	10	6.9	Prepubertal	NA	3.5	2D TA	Small subcapsular	Whole	In the ovarian stroma at the max distance from the surface
Battaglia et al. (b) [35]	2002	3	Prospective	15	7.6	Prepubertal	NA	3.5	2D TA	Small subcapsular	Whole	“
Battaglia et al. [36]	2006	3	Prospective	14	23.2	Reproductive age	NR	6.5	2D TV	Small antral	Whole	“
Battaglia et al. [37]	2012	3	Prospective	52	25.8	Reproductive age	NR	9.0	2D TV	Small subcapsular	Whole	“
Bentzen et al. [38]	2013	3	Retrospective	366	33.7	Reproductive age	Follicular	9.0	2D TV	2–10 mm	Whole	NA
Carmina et al. [3]	2005	3	Prospective	50	25.9	Reproductive age	Follicular	NR	2D TV	NA	NA	NR
Carmina et al. [39]	2018	3	Retrospective	28	23.4	Reproductive age	Follicular	10.0	2D TV	2–10 mm	Whole	NA
Catteau-Jonard et al. [40]	2012	3	Prospective	95	29.0	Reproductive age	Follicular	7.0	2D TV	2–9 mm	Whole	NA
Chan et al. [41]	2006	3	Retrospective	70	36	Reproductive age	NR	7.0	2D and 3D TV	< 10 mm	Whole	NA
Chen et al. (a) [42]	2008	3	Retrospective	26	17.85	Reproductive age	Follicular	6.0	2D TV	NA	NA	NA
Chen et al. (b) [43]	2008	3	Prospective	153	27.15	Reproductive age	Follicular	6.0	2D TV	All countable follicles	Whole	NA
Christ et al. [44]	2014	3	Retrospective	60	27	Reproductive age	Follicular	12.0	2D TV	2–10 mm	Whole	NA
Christiansen et al. [45]	2016	2	Cross-sectional	148	36.2	Reproductive age	Follicular	7.5	2D TV	2–9 mm	Whole	NA
Çil et al. [46]	2009	3	Prospective	25	33.04	Reproductive	Follicular	9.0	2D TV	2–10 mm	Whole	Small artery in the ovarian stroma not close to the surface
Codner et al. [47]	2006	3	Prospective	38	26.3	Reproductive age	NR	7.5	2D TA and TV	2–9 mm	Section	NA
Dao et al. (a) [48]	2019	3	Retrospective	55	7.5	Prepubertal	NA	NR	2D TA	NA	NA	NA
Dao et al. (b) [48]	2019	3	Retrospective	93	14.7	Reproductive age	NR	NR	2D TA	NA	NA	NA
De Guevara et al. [49]	2013	3	Prospective	35	37	Reproductive age	Follicular	7.5	2D TV	2–9 mm	Whole	NA
Deb et al. [50]	2013	3	Prospective	36	28.12	Reproductive age	Follicular	9.0	2D and 3D TV	2–10 mm	Whole	NA
Dewailly et al. [51]	2011	3	Retrospective	66	30	Reproductive age	Follicular	9.0	2D TV	< 10 mm	Whole	NA
Dumesic et al. [52]	2001	3	Cross-sectional	25	30.8	Reproductive age	Follicular	8.0	2D and 3D TV	< 10 mm	Whole	NA
Elgindy et al. [53]	2008	3	Prospective	33	30.19	Reproductive age	Follicular	6.5	2D TV	2–10 mm	Whole	NA
Erdem et al. [54]	2003	3	Retrospective	62	37.6	Reproductive age	Follicular	5.0	2D TV	< 8 mm	Whole	NA
Fruzzetti et al. [55]	2015	3	Cross-sectional	72	13.7	Reproductive age	Follicular	10.0	2D TA and TV	NA	NA	NA
Fulghesu et al. [5]	2001	3	Retrospective	30	NR	Reproductive age	Follicular	6.5	2D TV	NA	NA	NA
Fulghesu et al. [56]	2006	3	Prospective	10	24.4	Reproductive age	Follicular	6.5	2D TV	Total no. of follicles	Section	NA
Golestani et al. (a) [57]	2008	3	Retrospective	20	13.5	Reproductive age	NR	3.5	2D TA	NA	NA	NR
Golestani et al. (b) [57]	2008	3	Retrospective	40	9.6	Prepubertal	NA	3.5	2D TA	NA	NA	NR
Greenwood et al. [58]	2017	2	Prospective	226	33.1	Reproductive age	Follicular	8.0	2D TV	2–10 mm	Whole	NA
Herter et al. [59]	2002	3	Prospective	139	6	Prepubertal	NA	5.0	2D TA	NA	NA	NA
Homer et al. [60]	2019	2	Retrospective	20	25.7	Reproductive age	Follicular	9.0	3D TV	2–9 mm	Whole	NA
Jarrett et al. [61]	2020	2	Prospective	12	30	Reproductive age	Follicular	12.0	2D and 3D TV	NA	Whole	NA
Järvelä et al. (a) [62]	2002	3	Prospective	28	35	Reproductive age	Follicular	7.0	3D TV	2–8 mm	NA	NA
Järvelä et al. (b) [63]	2003	3	Prospective	29	NR	Reproductive age	Follicular	7.0	3D TV	Tot no. of follicles	Whole	NA
Järvelä et al. (c) [64]	2007	3	Prospective	11	30.8	Reproductive age	Follicular	7.0	3D TV	NA	NA	NA
Jokubkiene et al. [65]	2006	3	Prospective	14	28	Reproductive age	Follicular	10.0	3D TV	NA	NA	NA
Jokubkiene et al. [66]	2012	3	Prospective	214	30	Reproductive age	Follicular	12.0	3D TV	2–10 mm	Whole	NA
Jonard et al. [67]	2005	2	RCT	57	29	Reproductive age	NR	7.0	2D TV	2–9 mm	Whole	NA
Kline et al. [68]	2004	4	Prospective	65	35	Reproductive age	NR	10.0	2D TV	All countable follicles	Whole	NA
Köşüş et al. [69]	2011	3	Prospective	65	26.7	Reproductive age	Follicular	6.5	2D TV	All countable follicles	Whole	NA
Lam et al. [70]	2007	3	Prospective	40	32.5	Reproductive age	Follicular	7.5	3D TV	2–9 mm	Section	One of the main vessels with the ovarian stroma
Łebkowska et al. [71]	2016	2	cross-sectional	16	24	Reproductive age	Follicular	9.0	2D TV	2–9 mm	Whole	NA
Lie Fong et al. [72]	2017	3	Retrospective	297	28.3	Reproductive age	NR	7.5	2D TV	2–9 mm	Whole	NA
Lujan et al. [4]	2013	3	Prospective	70	27	Reproductive age	Follicular	12.0	2D TV	2–9 mm	Whole/section	NA
Merino et al. (a) [73]	2019	3	Retrospective	53	13.9	Reproductive age	Follicular	5.0	2D TA	2–9 mm	Section	NA
Merino et al. (b) [73]	2019	3	Retrospective	22	14.3	Reproductive age	Follicular	5.0	2D TA	2–9 mm	Section	NA
Murphy et al. [74]	2006	3	Prospective	17	29.7	Reproductive age	Follicular	5.0	2D TV and TA	2–10 mm	Section	NA
Orbak et al. [75]	2007	3	Retrospective	55	0.01	Prepubertal	NA	7.5	2D TA	NA	NA	NA
Özay et al. [76]	2019	3	Prospective	90	21.54	Reproductive age	NR	9.0	2D TV	NA	NA	In the ovarian stroma at the max distance from the surface
Ozkan et al. [77]	2007	3	Prospective	43	20.8	Reproductive age	Follicular	6.0	2D TV	NA	NA	Ovarian stroma and in the wall of dominant follicle or c.l
Pan et al. [78]	2002	3	Prospective	100	30.9	Reproductive age	NR	NR	3D TV	NA	NA	NA
Panidis et al. [79]	2012	3	Prospective	254	31.3	Reproductive	NR	NR	2D TV	2–9 mm	Whole	NA
Pascual et al. [80]	2008	3	Retrospective	45	32.3	Reproductive age	Follicular	10.0	3D TV	2–9 mm	Whole	NA
Peigné et al. [81]	2018	3	Case–control	157	29	Reproductive age	Follicular	9.0	2D TV	2–9 mm	Whole	NA
Pellizzari et al. [82]	2002	3	Case–control	13	24.15	Reproductive age	Follicular	6.5	2D TV	NA	NA	Within the ovarian stroma
Petri Nahás et al. [83]	2004	3	Prospective	30	36.66	Reproductive age	NR	7.5	2D TV	NA	NA	Ovarian artery in the pelvic infundibulum
Phy et al. [84]	2004	4	Prospective	33	30.4	Reproductive age	Follicular	8.0	2D and 3D TV	2–10 mm	Section	NA
Pirgon et al. [85]	2015	3	Case–control	30	15.2	Reproductive age	Follicular	7.5	2D TV	2–10 mm	Whole	NA
Pirwany et al. [86]	2001	4	cross-sectional	14	31.2	Reproductive age	Follicular	NR	NR	NA	NA	NA
Rosenfield et al. [87]	2012	3	Prospective	19	24.5	Reproductive age	Follicular	9.0	2D TA and TV	2–9 mm	NA	NA
Santoro et al. (a) [88]	2003	4	Prospective	14	NR (> 45)	Reproductive age	Follicular	5.0	2D TV	< 10 mm	Whole	NA
Santoro et al. (b) [88]	2003	4	Prospective	22	NR	Reproductive age	Follicular	5.0	2D TV	< 10 mm	Whole	NA
Sanverdi et al. [89]	2018	4	Cross-sectional	139	31.04	Reproductive age	Follicular	NR	2D TV	AFC	Whole	NA
Sasaki et al. [90]	2019	3	Retrospective	118	32.66	Reproductive age	Follicular	7.0	NR	AFC	Whole	NA
Scheffer et al. (a) [91]	2003	3	Prospective	49	NR	Reproductive age	Follicular	7.5	2D TV	2–10 mm	Whole	NA
Scheffer et al. (b) [91]	2003	3	Prospective	53	NR	Reproductive age	Follicular	7.5	2D TV	2–10 mm	Whole	NA
Scheffer et al. (c) [91]	2003	3	Prospective	60	NR	Reproductive age	Follicular	7.5	2D TV	2–10 mm	Whole	NA
Shahrami et al. [92]	2016	4	Cross-sectional	53	27.23	Reproductive age	Follicular	NR	NR	NA	NA	NA
Shen et al. [93]	2008	3	Prospective	23	31	Reproductive age	Follicular	9.0	2D TV	NA	NA	Large vessel at ovarian hilum
Singha et al. [94]	2015	4	Cross-sectional	52	37	Reproductive age	NR	8.0	2D TV	NA	NA	NA
Su et al. [95]	2008	4	Cross-sectional	18	45	Reproductive age	Follicular	NR	2D TV	2–10 mm	Whole	NA
Taponen et al. [96]	2004	2	Retrospective	58	31	Reproductive age	NR	6.0	2D TV	2–8 mm	Section	NA
Tomioka et al. [97]	2018	3	Prospective	11	30	Reproductive age	Follicular	10.0	2D TA and TV	2–10 mm	Whole	NA
van Hooff et al. [98]	2000	3	Prospective	58	16.4	Reproductive age	Follicular	5.0	2D TA	NA	NA	NA
Weerakiet et al. [99]	2007	3	Cross-sectional	21	33.81	Reproductive age	Follicular	7.5	2D TV	< 10 mm	Whole	NA
Wongwananuruk et al. [100]	2018	3	Prospective	63	29.7	Reproductive age	Follicular	8.0	2D TV or TR	2–9 mm	Whole/section	NA
Younis et al. [101]	2011	3	Prospective	101	28.76	Reproductive age	Follicular	9.0	2D TV	2–9 mm	Whole	Stromal ovarian blood flow
Zhang et al. [102]	2013	3	Case–control	685	26.56	Reproductive age	NR	NR	2D TV	2–9 mm	Whole	NA

*LoE* level of evidence according to the Oxford Centre for Evidence-Based Medicine, *NA* not applicable, *NR* not reported, *PI* pulsatilty index, *TA* transabdominal, *TV* transvaginal, *2D* two-dimensional, *3D* three-dimensional

### Statistical analysis

Four separate meta-analyses were conducted, one for every outcome (ovarian volume, ovarian follicular count—whole ovary, ovarian follicular count—ovarian section, ovarian artery PI). Heterogeneity was assessed using *I*^2^ statistics and a random-effects model was applied for all analyses. The effect measures were expressed as mean [lower limit; upper limit]. When the mean was not provided, the closest approximation of mean and SD (standard deviation) from median and IQR (interquartile range) was calculated [23].

Subgroup analyses were performed: (a) according to 5 age bands in studies including reproductive-aged women: < 20 years old (1st group); ≥ 20 and < 25 years old (2nd group); ≥ 25 and < 30 years old (3rd group); ≥ 30 and < 35 years old (4th group); and ≥ 35 years old (5th group); (b) according to 4 groups identified with the upper limit of MHz reported for the transducer: ≤ 5 MHz (1st group), 5–7.5 MHz (2nd group), 8–9 MHz (3rd group), and 10–12 MHz (4th group). Meta-regression analyses were conducted in order to test the effects of age on the evaluated parameters.

All analyses were performed using Comprehensive Meta-analysis Version 2, Biostat (Englewood, NJ, USA).

## Results

### Analysis of available studies

**Fig. 1:**
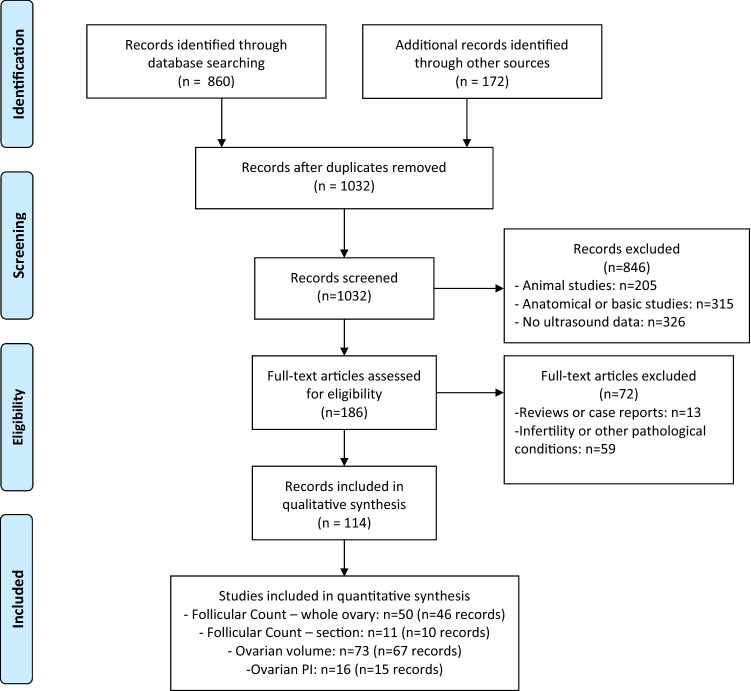
Flow diagram for the studies included in the meta-analyses. From PRISMA 2009 flow diagram. From reference [103]

The initial literature search produced 1032 potentially relevant citations. After screening and detailed assessment (see flow chart—Fig. 1 for reasons for exclusion), 50 studies were included in the meta-analysis of follicular count in the whole ovary, 11 in the meta-analysis of follicular count per ovarian section, 73 in the meta-analysis of ovarian volume, and 16 in the meta-analysis of ovarian PI. Some papers provided data for more than one of the 4 meta-analyses. The flow chart of study selection is reported in Fig. 1 and the details of the retrieved studies are reported in Table 1.

To determine the age-related differences in ovarian parameters, studies enrolling pre-pubertal girls or fertile women were considered separately. Data on menopausal women were not sufficient to perform a meta-analysis. Moreover, in the analysis of the studies including reproductive-aged women, when information on age was available, the results were stratified into 5 age bands: < 20 years old (1st group); ≥ 20 and < 25 years old (2nd group); ≥ 25 and < 30 years old (3rd group); ≥ 30 and < 35 years old (4th group); and ≥ 35 years old (5th group).

### Ovarian follicular count: whole ovary

Studies reporting information on follicular count calculated on the whole ovary were included in a separate meta-analysis from those reporting the same data obtained within an ovarian section. In general, we included studies reporting information on all visible follicles measuring ≥ 2.0 mm, with an upper limit of 8.0–10.0 mm (see Table 1).

**Fig. 2:**
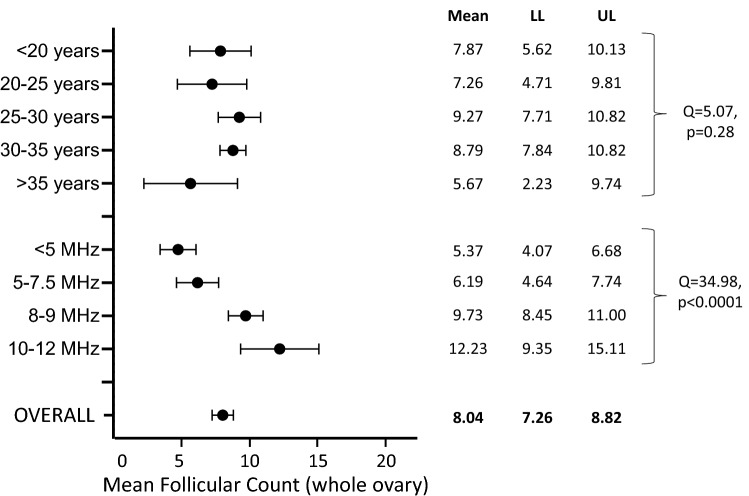
Mean follicular count (whole ovary) at ultrasound in healthy women of reproductive age. *LL* lower limit, *UL* upper limit

Fifty studies were identified including information on follicular count calculated on the whole ovary. After excluding the 2 studies enrolling pre-pubertal girls [35a and b], the overall mean follicular number was 8.04 [7.26–8.82] (*n* = 5013 subjects, mean age 29.66 years; Fig. 2).

After stratifying the population in reproductive years according to the above-mentioned age groups, a mean follicular count of 7.87 [5.62–10.13] was found in the 1st group (< 20 years; *n* = 2 studies) [29, 85], 7.26 [4.71–9.81] in the 2nd group (≥ 20 and < 25 years; *n* = 4 studies) [3, 36, 39, 71], 9.27 [7.71–10.82] in the 3rd group (≥ 25 and < 30 years; *n* = 14 studies) [4, 37, 40, 43, 44, 50, 67, 69, 72, 81, 100–102], 8.79 [7.84–9.74] in the 4th group (≥ 30 and < 35 years; *n* = 16 studies) [27, 28, 38, 46, 51–53, 58, 61, 66, 79, 80, 89, 90, 97, 99], and 5.67 [2.23–9.12] in the 5th group (≥ 35 years; *n* = 9 studies) [41, 45, 49, 54, 68, 88a, 91b and c, 95], respectively (Fig. 2). Overall, among the 5 groups, no statistically significant difference in ovarian follicular count appeared (*Q* = 5.07, *p* = 0.28) (Fig. 2). However, at meta-regression analysis, age showed a significant modulation effect in reproductive-aged women on follicular count calculated on the whole ovary (*n* = 42 studies) (*s* = 21.63, *p* < 0.0001; *I* = 6.93, *p* < 0.0001].

We also explored the differences in the follicular count according to the frequency of the transducer. Considering the upper limit of MHz reported for the transducer in each study, 4 groups were identified: ≤ 5 MHz (1st group), 5–7.5 MHz (2nd group), 8–9 MHz (3rd group), and 10–12 MHz (4th group). A mean follicular count of 5.37 [4.07–6.68] was found in the 1st group (4 studies) [34, 54, 88a and b], 6.19 [4.64–7.74] in the 2nd group (18 studies) [27, 36, 40, 41, 43, 45, 49, 53, 63, 67, 69, 72, 85, 90, 91a–c, 99], 9.73 [8.45–11.00] in the 3rd group (15 studies) [ 28, 29, 37, 38, 39, 46, 50–52, 58, 60, 71, 81, 100, 101], and 12.23 [9.35–15.11] in the 4th group (7 studies) [4, 44, 61, 66, 68, 80, 97] (Fig. 2). Four studies failed to provide information on this parameter. A significant difference was found when stratifying the studies according to the transducer’s MHz (*Q* = 34.98, *p* < 0.0001) (Fig. 2). Therefore, pairwise comparisons of studies in the 4 groups were performed, and a statistically significant difference was found in the follicular count:

- between the 1st and the 3rd groups (≤ 5 vs. 8–9 MHz) [*Q* = 21.86; *p* < 0.0001] and between the 1st and the 4th groups (≤ 5 vs. 10–12 MHz) [*Q* = 18.07; *p* < 0.0001];
- between the 2nd and the 3rd groups (5–7.5 vs. 8–9 MHz) [*Q* = 11.95; *p* = 0.001] and between the 2nd and the 4th groups (5–7.5 vs. 10–12 MHz) [*Q* = 13.11; *p* < 0.0001].

### Ovarian follicular count: a section

**Fig. 3:**
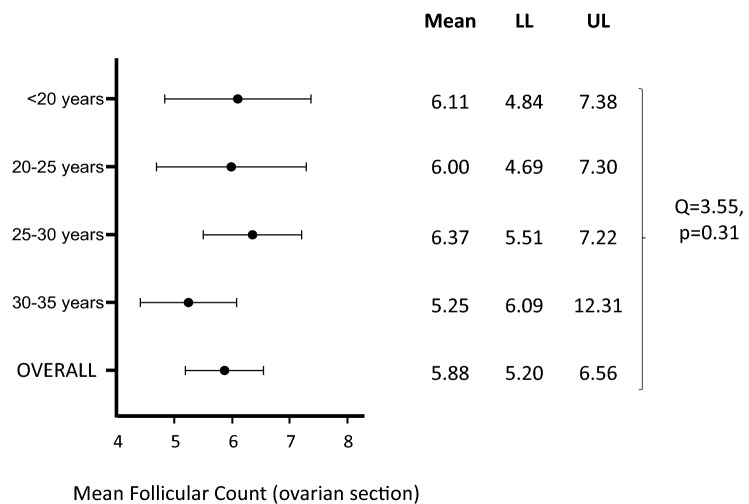
Mean follicular count (within ovarian section) at ultrasound in healthy women of reproductive age. *LL* lower limit, *UL* upper limit

Eleven studies including information on ovarian follicular count calculated within an ovarian section (maximum length in sagittal section) were identified. All of them considered women in their reproductive years. The pooled ovarian follicular number was 5.88 [5.20–6.56] (Fig. 3).

After stratifying the studies according to the previously identified age groups, no studies were found in the 5th one (≥ 35 years). Mean ovarian follicular counts of 6.11 [4.84–7.38], 6.00 [4.69–7.30], 6.37 [5.51–7.22] and 5.25 [6.09–12.31] ml were found in the 1st (< 20 years; *n* = 2 studies) [73a and b], 2nd (≥ 20 and < 25 years; *n* = 1 study) [56], 3rd (≥ 25 and < 30 years; *n* = 4 studies) [4, 47, 74, 100], and 4th group (≥ 30 and < 35 years; *n* = 4 studies) [28, 70, 84, 96], respectively, with no statistically significant difference between the four groups (*Q* = 3.55, *p* = 0.31) (Fig. 3). Similarly, no significant difference was found when stratifying the studies according to the transducer’s MHz (*Q* = 2.73, *p* = 0.43) (not shown).

### Ovarian volume

**Fig. 4:**
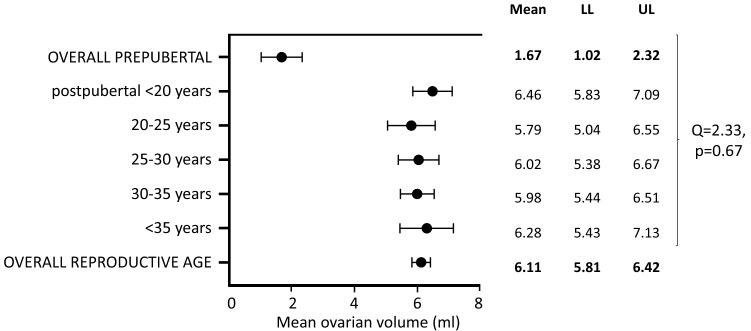
Mean ovarian volume (ml) at ultrasound, calculated using the formula for a prolate ellipsoid, in healthy pre-pubertal girls and women of reproductive age. *LL* lower limit, *UL* upper limit

Seventy-three studies were identified, which reported information on ovarian volume calculated using the formula for a prolate ellipsoid. Among them, 65 studies enrolled women in their reproductive years, whereas 6 [31, 35a and b, 48a, 57b, 59] enrolled girls of pre-pubertal age, one newborn [75] and one post-menopausal woman [30]. When considering the 65 studies including women in their reproductive years (*n* = 4107 subjects, mean age 27.42 years), the overall mean ovarian volume was 6.11 [5.81–6.42] ml (Fig. 4). After excluding one study enrolling newborns [75], in the remaining six studies considering subjects of pre-pubertal age (*n* = 358 subjects, mean age 7.42 years), the mean ovarian volume was 1.67 [1.02–2.32] ml (Fig. 4), which was significantly lower than in women in reproductive age (*Q* = 147.05, *p* < 0.0001).

Among the studies on women in their reproductive years, 62 provided information on the mean age or the age range of the sample. We analyzed the studies according to the above-mentioned 5 age sub-groups, and found a mean ovarian volume of 6.46 [5.83–7.09] ml in the 1st group (< 20 years; *n* = 9 studies) [29, 42, 48b, 55, 57a, 73a and b, 85, 98], 5.79 [5.04–6.55] ml in the 2nd group (≥ 20 and < 25 years; *n* = 9 studies) [24, 34, 36, 39, 56, 71, 77, 82, 87], 6.02 [5.38–6.67] ml in the 3rd group (≥ 25 and < 30 years; *n* = 17 studies) [3, 4, 25, 26, 37, 43, 44, 47, 50, 65, 67, 69, 74, 92, 100–102], 5.98 [5.44–6.51] ml in the 4th group (≥ 30 and < 35 years; *n* = 18 studies) [28, 38, 46, 51–53, 61, 64, 66, 70, 78–80, 84, 86, 96, 97, 99], and 6.28 [5.43–7.13] ml in the 5th group (≥ 35 years; *n* = 9 studies) [41, 49, 54, 62, 83, 91b and c, 94, 95], respectively (Fig. 4). Overall, among the 5 groups in reproductive age, no statistically significant difference in ovarian volume was found (*Q* = 2.33, *p* = 0.67) (Fig. 4). However, at meta-regression analysis, exploring the effect of age on ovarian volume in reproductive-aged women (*n* = 60 studies), a significance relationship was observed [slope (*s*) 0.01, *p* = 0.06; intercept (*I*) 5.04, *p* < 0.0001].

### Ovarian pulsatility index (PI)

**Fig. 5:**
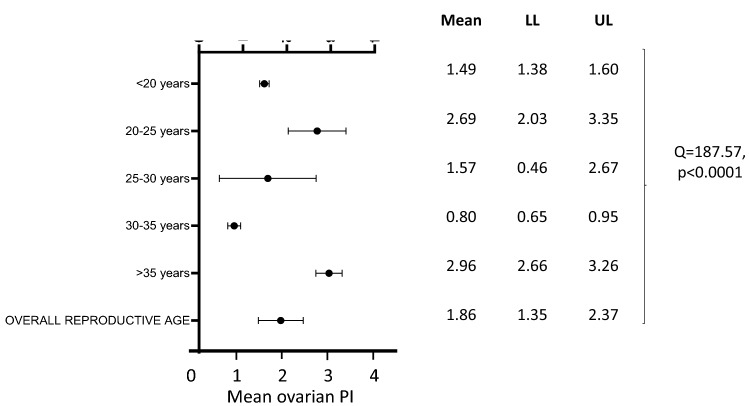
Mean ovarian pulsatility index (PI) at Doppler ultrasound in healthy women of reproductive age. *LL* lower limit, *UL* upper limit

Sixteen studies including information on ovarian artery PI were identified (see Table 1). After excluding two studies enrolling pre-pubertal girls [35a, 57a], which used a transabdominal approach, the pooled mean PI was 1.86 [1.35–2.37] (Fig. 5). All studies except one [70] employed a two-dimensional (2D) technique.

After stratifying the studies according to the previously identified age bands, a mean ovarian PI of 1.49 [1.38–1.60], 2.69 [2.03–3.35], 1.57 [0.46–2.67], 0.80 [0.65–0.95], and 2.96 [2.66–3.26] was found in the 1st (< 20 years; *n* = 1 study) [57b], 2nd (≥ 20 and < 25 years; *n* = 5 studies) [24, 36, 76, 77, 82], 3rd (≥ 25 and < 30 years; *n* = 3 studies) [3, 37, 101], 4th (≥ 30 and < 35 years; *n* = 4 studies) [33, 46, 70, 93], and 5th groups (≥ 35 years; *n* = 1 study) [83], respectively, with a statistically significant difference between the five groups (*Q* = 187.57, *p* < 0.0001) (Fig. 5). Therefore, pairwise comparisons of groups with different mean age bands were performed, and a statistically significant difference was found in ovarian PI:

- between the 1st and the 2nd groups (< 20 vs. ≥ 20 and < 25 years) [*Q* = 12.42; *p* < 0.0001], between the 1st and the 4th groups (< 20 vs. ≥ 30 and < 35 years) [*Q* = 54.49; *p* < 0.0001], between the 1st and the 5th groups (< 20 vs. ≥ 35 years) [*Q* = 82.83; *p* < 0.0001];
- between the 2nd and the 4th groups (≥ 20 and < 25 vs. ≥ 30 and < 35 years) [*Q* = 30.15, *p* < 0.00001];
- between the 3rd and the 5th groups (≥ 25 and < 30 vs. ≥ 35 years) [*Q* = 5.73, *p* = 0.017];
- between the 4th and the 5th groups (≥ 30 and < 35 vs. ≥ 35 years) [*Q* = 163.27, *p* < 0.0001] (Fig. 5).

Twelve studies on women in their reproductive years provided information on the transducer’s frequency; no studies were identified in the 4th group (10–12 MHz). No significant difference on ovarian artery PI emerged when stratifying the studies according to the different MHz (*Q* = 4.90, *p* = 0.09) (not shown).

## Discussion

The present systematic and meta-analytic approach demonstrates for the first time that the overall mean ovarian volume was 6.11 ml in women in reproductive age and 1.67 ml in pre-pubertal girls, with a range of 5.81–6.42 and of 1.02–2.32, respectively. In reproductive age, the overall mean follicular count was 8.04 when calculated in the whole ovary and 5.88 when calculated in an ovarian section, with a range of 7.26–8.82 and of 5.20–6.56, respectively. However, age and the frequency of the transducers were found to significantly modulate these values. In contrast, the authors agreed that all the other sonographic parameters (i.e., ovarian stroma) could not be considered eligible for meta-analysis, mainly due to the paucity and heterogeneity of data.

The standardization of ultrasound parameters is of paramount clinical relevance since it contributes to the diagnostic workflow of several endocrine conditions, including Premature Ovarian Insufficiency, PCOS and poor ovarian responders (POR) in ART procedures (Bologna criteria: antral follicle count ≤ 5–7 follicles [104]. Noteworthy, we hereby demonstrated that the population of women with a normal ovarian function showed a follicular count ranging between 7.26 and 8.82, thus corroborating the proposed criteria for either PCOM (> 12 according to Rotterdam criteria) or POR (≤ 5–7 according to Bologna criteria).

Another relevant finding of the present study is the apparent age-dependent modulation of the total ovarian follicular count (as calculated in the whole ovary). Despite being not able to highlight a significant difference among the 5 age sub-groups, we noted that the age range 25–30 years was the one with the higher mean follicular count (9.27), followed by a progressive age-related reduction (5.67 in fertile women older than 35 years). In addition, pubertal girls younger than 20 showed a higher mean follicular count (7.87) than those in the 20–25-year group (7.26). In line with this trend, multifollicular ovaries are seen commonly in girls with a gynecological age of ˂ 8 years, and should be considered as a physiologic condition during early adolescence [105]. This is relevant to avoid a misdiagnosis of PCOS in this population. A similar age-modulated trend, although without significant differences between groups, was observed when the follicles were counted in an ovarian section. Our study, meta-analyzing data on follicular count obtained in different ultrasound settings, could be of relevance since the analysis of follicular count per ovarian section is commonly performed in clinical practice.

Another important parameter that we were able to meta-analyze was the ovarian volume. As expected, a significant difference was found when comparing pre-pubertal girls and women in their reproductive years (mean values 1.67 vs. 6.11, respectively), whereas our data did not reveal a clear modulation by age in fertile women. As observed for the follicular count, even when we considered the mean upper limit for normal ovarian volume (6.42 ml), this value fell well below the proposed PCOM threshold of 10 ml, thus supporting the appropriateness of this cut-off in defining PCOM.

Even though the ovarian PI is a Doppler ultrasound parameter with a more limited clinical application, we were able to meta-analyze available data while finding again an age-dependent modulation, which revealed two peaks: one pertaining the 20–25 years and one pertaining the > 35 years group. Intriguingly, PI of arterial blood vessels within the genital district has been previously reported to increase as a function of metabolic risk factors, including body mass index, waist circumference, and insulin-resistance biomarkers [106]. However, since data on cardiometabolic risk factors or relative treatments were not systemically available in the included studies, the potential contributions of these mediators could only be mentioned without any inference.

Regarding the role of ultrasound methodology, the TV approach has been proved as more sensitive and specific than the TA one, not only in the diagnosis of pelvic disease of gynecologic origin, but also in cases of ovarian follicle monitoring and evaluation for PCOM [107]. In the 2014 “task force report from the Androgen Excess and Polycystic Ovary Syndrome Society”, which proposed a threshold of ≥ 25 follicle number per ovary to define PCOM when using transducers with a frequency ≥ 8 MHz, the TA route was described as “not suitable for recording a precise follicle count” [6]. It should be noted that, in situations when the TA approach is the only possible, it can provide a reliable assessment of ovarian volume. As for the transrectal route, in adolescent patients, a 3D version combined with the TA technique has been showed to improve the precision of PCOM definition [108]. In the reviewed studies, all those performed in pre-pubertal girls used a TA-only approach, whereas none reported the use of the transrectal one. Regarding the studies on women in reproductive age, only 4 employed only TA ultrasound [48, 57, 73, 98], while a very few others [25, 29, 47, 55, 74, 87, 97] reported the use of both methods (TA and TV) (see Table 1). None of the 4 studies conducted with TA ultrasound provided data for the meta-analysis of Ovarian Follicular Count in the whole ovary, and only one [73] provided data for the meta-analysis of Ovarian Follicular Count per section. Therefore, no subgroup analysis was performed.

The main strength of our research is the standardized, meta-analytic approach, which, to our knowledge, is the first ever conducted on this topic. In addition, we decided to perform several sub-analyses according to potential modulating factors, including follicular count obtained per ovarian section, which is commonly performed in clinical practice. In addition, we refrained from interpreting our findings in a pathological setting. The key guidance of the present document was to provide informative and user-friendly data, that could be used and critically considered in future research on pathological ovarian conditions. It is advisable that the much-needed standardization of ovarian ultrasonography will allow the development of more evidence-based, universally accepted criteria for the diagnosis of ovarian disorders, especially PCOM.

Among the limitations, we would like to mention the lack of an indication pertaining the cycle phase for ovarian ultrasound; however, ad highlighted in the summarizing Table, the vast majority of records reported to have conducted the ultrasound study during the follicular phase.

## Conclusions

In conclusion, our systematic review and meta-analysis provides a relevant clinical information for a more accurate assessment of physiological ultrasound ovarian parameters in pre-pubertal girls and women in reproductive age. Each center should standardize ovarian US according to the available machines, at least when analyzing those parameters which resulted to be significantly modulated by the quality of the transducer. However, we strongly believe that such guidance should improve the interpretation and diagnostic accuracy of ovarian ultrasound parameters in different physiological and pathological settings.
